# Upregulation of lncRNA147410.3 in the Brain of Mice With Chronic *Toxoplasma* Infection Promoted Microglia Apoptosis by Regulating Hoxb3

**DOI:** 10.3389/fncel.2021.648047

**Published:** 2021-05-18

**Authors:** Yongliang Wang, Ruxia Han, Zhejun Xu, Xiahui Sun, Chunxue Zhou, Bing Han, Shenyi He, Hua Cong

**Affiliations:** ^1^Department of Pathogenic Biology, School of Basic Medical Sciences, Cheeloo College of Medicine, Shandong University, Jinan, China; ^2^College of Animal Science and Technology, Jilin Agricultural University, Changchun, China

**Keywords:** *Toxoplasma gondii*, lncRNA147410.3, microglia, Hoxb3, apoptosis

## Abstract

*Toxoplasma gondii* is neurotropic and affects the function of nerve cells, while the mechanism is unclear. LncRNAs are abundantly enriched in the brain and participated in the delicate regulation of the central nervous system (CNS) development. However, whether these lncRNAs are involved in the regulation of microglia activation during the process of *T. gondii* infection is largely unknown. In this study, the upregulation of a novel lncRNA147410.3 (ENSMUST00000147410.3) was identified as a key factor to influence this process. The target gene of lncRNA147410.3 was predicted and identified as Hoxb3. The localization of lncRNA147410.3 in the brain and cells was proved in the nucleus of neuroglia through FISH assay. Furthermore, the function of lncRNA147410.3 on neuronal cell was confirmed that lncRNA147410.3 could affect proliferation, differentiation, and apoptosis of mouse microglia by positively regulating Hoxb3. Thus, our study explored the modulatory action of lncRNA147410.3 in *T. gondii* infected mouse brain, providing a scientific basis for using lncRNA147410.3 as a therapeutic target to treat neurological disorder induced by *T. gondii*.

## Introduction

*Toxoplasma gondii* can infect almost all nucleated cells in warm blooded animals, including humans. Approximately, 30% of the population is infected with *T. gondii* latently all over the world (Montoya and Liesenfeld, 2004). *T. gondii* is neurophilic and can induce *Toxoplasma* encephalitis (Frénal et al., 2017). However, the mechanism that associated with *Toxoplasma* encephalitis are unclear.

*Toxoplasma* tachyzoites invade and replicate in host nucleated cells actively (Caldas et al., 2016). The tachyzoites can utilize the migration of immune cells (Lambert et al., 2006) to enter the blood circulation and reach the central nervous system (CNS; Lambert et al., 2009). The tachyzoite can infect microglia, astrocytes and neurons *in vitro* (Scheidegger et al., 2005). *T. gondii* alters the behavior of its hosts when the infection is in the latent phase, which is characterized by the parasitic cysts in the brain (David et al., 2016). Infected rodents exhibit impaired learning and memory, as well as enhanced activities (Naruse et al., 2018). In addition, *Toxoplasma* infection has been closely associated with a number of psychiatric disorders. A number of studies have shown that *Toxoplasma* infection is positively correlated with the occurrence of schizophrenia, epilepsy, neurodegenerative diseases such as Parkinson’s and Alzheimer’s disease (Tyebji et al., 2019). There is a controversy regarding the role of *T. gondii* on astrocytes and microglia, which may act as a receptor for parasite proliferation or as a protective immune response activator within the central nervous system.

With the development of high throughput sequencing, many novel non-coding RNAs (ncRNAs) have been identified. Long stranded non-coding RNAs (lncRNAs) are a class of non-coding RNA molecules with a transcription length greater than 200 nucleotides, which display typically more tissue specificity than protein coding genes (Aprea et al., 2013; Luo et al., 2013). It is found that lncRNAs are enriched in the brain. Spatial and temporal regulation of lncRNAs are hypothesized to promote neuronal diversity and specificity. Many studies have shown that lncRNAs are involved in mechanisms of neuronal action (Heinen et al., 2013; Yao et al., 2018). However, the role of lncRNAs in the brain of *Toxoplasma* infected mice was unclear.The relationship between lncRNA and *T. gondii* encephalopathy remains to be explored. Therefore, we selected BV-2 cells to explore the role of lncRNAs in microglial cells during *T. gondii* chronic infection.

In this study, a mice model of chronic *Toxoplasma* type II PRU strain infection was established. Gene microarray results showed that lncRNA147410.3 was significantly upregulated in the brain of infected mice. The target gene of lncRNA was predicted to be the homologous cassette gene Hoxb3, which is a family of Hoxb3 that encodes transcription factors and regulates neuronal differentiation and proliferation during embryogenesis and normal development (Gray et al., 2011). However, the relationship between lncRNA147410.3 and Hoxb3 in the brain of *T. gondii* infected mice is unclear. Thus, the present study proposes to investigate this regulatory mechanism to reveal that the molecular mechanisms of *T. gondii* on brain damage which will provide strategies for the early intervention of *Toxoplasma* encephalitis.

## Materials and Methods

### Parasites

*T. gondii* PRU strain, a low virulent type II strain, was kindly gifted from Prof. Jilong Shen and passaged in our laboratory. A chronic *T. gondii* infected BALB/c mouse model was established *via* administration by gavage at a dose of 20–30 cysts. All experiments in the present study were performed 8 weeks after infection.

### Mice and Ethical Approval

Female BALB/c mice (6–8 weeks old) were delivered from Shandong University Experimental Animal Center. The mice were housed five per cage under pathogen free conditions and were adequately supplied with sterilized water and food. The breeding condition and experimental procedures were in strict accordance with the Code of Ethics for Experimental Animals of Shandong University.

### Cell Culture

The microglia (BV-2 cells) were passaged and frozen in our laboratory. BV-2 cells were cultured in Dulbecco’s modified Eagle’s medium (DMEM, HyClone, Logan, UT, USA) containing 10% fetal bovine serum and 1% Penicillin-Streptomycin solution (Beyotime, China) at 37°C in an incubator with 5% CO_2_.

### Plasmids Construction

The short hairpin RNAs (shRNA) interference system was constructed using plasmid pGPU6/GFP/Neo. The construct sequence of sh-RNA147410.3 was shown as 5′-CACCGGACTGGAAACGGTCTGTTTGTTCAAGAGACAAACAGACCGTTTCCAGTCCTTTTTTG-3′. oe-lncRNA147410.3 was constructed with pCDNA3.1(+) to overexpress lncRNA147410.3.

### Cell Transfection

The cells were transfected with plasmids (sh-lncRNA147410.3 and oe-lncRNA147410.3) when the cell density reaches 60%–80%. Lipofectamine 3000 (Invitrogen) was used for the transfection of plasmids. The efficiency of silencing or overexpression of lncRNA147410.3 was measured by qRT-PCR 48 h after transfection.

### Quantitative Real-Time PCR (qRT-PCR) Assay

Total RNA was extracted from mouse brain or cell lines use EASYspin Plus Tissue/Cell RNA Rapid Extraction Kit according to instructions (Aidlab, China). Total RNA was reverse transcribed strictly according to the HiScript^®^II QRT SuperMix for qPCR (^+^gDNA wiper) kit (Vazyme, China). Real-time quantitative PCR (qRT-PCR) was performed in strict accordance with the ChamQTM SYBR^®^ qPCR Master Mix kit (Vazyme, China).

The Ct values were obtained from the qPCR amplification curves, and the expression ratios of lncRNAs compared to the internal reference were calculated using the 2^−ΔΔCt^ algorithm with snRNA U6 or GAPDH as the internal reference. The primer sequences were as below, lncRNA147410.3 (F 5′-TGTGTTTCATGCGTCGGTTC-3′, R 5′-ACCATCATTGGGTCTGTGCC-3′), Hoxb3 (F 5′-TTCCAGAACCGTCGCATGAA-3′, R 5′-GGGGTCATGGAGTGTAAGGC-3′), GAPDH (F 5′-GGACACTGAGCAAGAGAGGC-3′, R 5′-TTATGGGGGTCTGGGATGGA-3′).

### CCK-8 Assay

To evaluate the proliferation of BV-2 cells, the cells were counted by Cell Counting Kit (CCK-8, Dojindo, Japan). The 96-well plate was seeded with 100 μl of BV-2 cell suspension, and was incubated in a 5% CO_2_ incubator at 37°C. When the cells grew to 70–80%, cells were transfected with interference and overexpression plasmids by Micropoly-transfecter™ Cell Reagent. After 12, 24, 36, 48, and 60 h of incubation, 10 μl of CCK-8 reagent and 100 μl of fresh DMEM cell medium were added to each well and incubated at 37°C for 1–2 h. Subsequently, the absorbance value was detected at 450 nm by the Automatic Enzyme Label Analyzer (Allsheng, China). Each experiment was performed in triplicate and repeated three times to ensure accuracy.

### Cell Cycle Analyses

BV-2 cells were collected 48 h after transfection of plasmids. The cells were added to pre-cooled (−20°C) 75% ethanol and mixed overnight at 4°C. The cells were washed with PBS and treated with RNase, followed by staining with PI solution (Becton, Dickinson and Company) and incubation at 37°C for 1 h. The cells labeled by fluorescent are detected by Flow Cytometer. Subsequently, the cell cycle was assayed using Modfit software.

### RNA FISH Assay

Fluorescence binding of lncRNA147410.3 probes were used for fluorescence *in situ* hybridization (FISH) analysis. *T. gondii* infected brain slide and BV-2 cells were stained with lncRNA147410.3 probes, and the staining procedure was done according to RNA FISH *in situ* hybridization kit (GenePharma). The location of the target lncRNA147410.3 in BV-2 cells and Brain tissues can be directly observed by Zeiss fluorescence confocal microscopy or MIDI panoramic scanner.

### TUNEL Assay

BV-2 cells were seeded on coverslips in 6-well plates, transfected with the mixture of Lipo 3000 cell transfection reagent and shRNA or overexpression plasmid. The cells were fixed with 4% paraformaldehyde at 37°C for 20 min after 48 h. Cell apoptosis was detected using TUNEL assay kit (Beyotime, China) according to the manufacturer’s instructions.

### Data Analysis

The experimental data were analyzed by *t*-test and analysis of variance (ANOVA), and statistical analysis was performed with GraphPad Prism 7.0 software, and data *p* < 0.05 indicated statistical significance.

## Results

### The Expression of LncRNA147410.3 in the *T. Gondii* Infected Mice Brain

**Figure 1:**
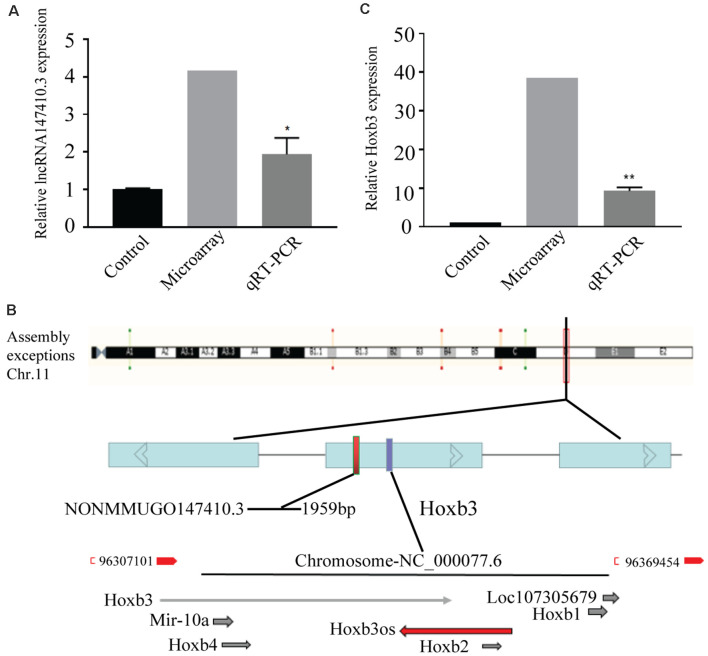
The expression of lncRNA147410.3 in the *Toxoplasma gondii* (*T. gondii*) infected mice brain and the prediction of its target gene. **(A)** The expression of lncRNA147410.3 was tested in the mice brain infected with *T. gondii* by microarray and quantitative polymerase chain reaction (qPCR). **(B)** Prediction the location of the target gene of lncRNA147410.3. **(C)** The expression of Hoxb3 in the infected mice brain. All values are presented as mean ± SD for three independent experiments. *p < 0.05, **p < 0.01, Student’s *t*-test.

The lncRNA147410.3 expression in the brain of *T. gondii* infected mice and uninfected mice was detected by gene microarray. The result indicated that the expression levels of lncRNA147410.3 were significantly increased in the brain of *T. gondii* infected mice than uninfected mice. Furthermore, qRT-PCR was applied to quantify the lncRNA in the brain of mice infected with the *T. gondii* PRU strain. The results showed that the expression level of lncRNA147410.3 in the brain of *T. gondii* infected mice 45 days later was as twice as that in the brain of uninfected mice (Figure 1A).

### The Prediction and Expression of Target Genes of LncRNA147410.3

The chromosome location of lncRNA147410.3 was confirmed through the UCSC Genome Browser. lncRNA147410.3 is located on the mouse chromosome 11 and its transcript length is 1,959 bp. Based on the expression correlation and the genome positional proximity of lncRNA and mRNA Hoxb3 was predicted as the potential target gene of lncRNA147410.3 by *cis/trans* analysis (Figure 1B).

The expression of lncRNA147410.3 and Hoxb3 in the brain was detected by gene microarray and qPCR. The result indicated that the expression of Hoxb3 in infected mice increased 10-fold compared with uninfected mice, which indicated that the elevated expression of Hoxb3 induced by chronic infection, and also validated the results of gene microarray (Figure 1C).

### The Regulation of the Target Gene Hoxb3 by LncRNA147410.3

**Figure 2:**
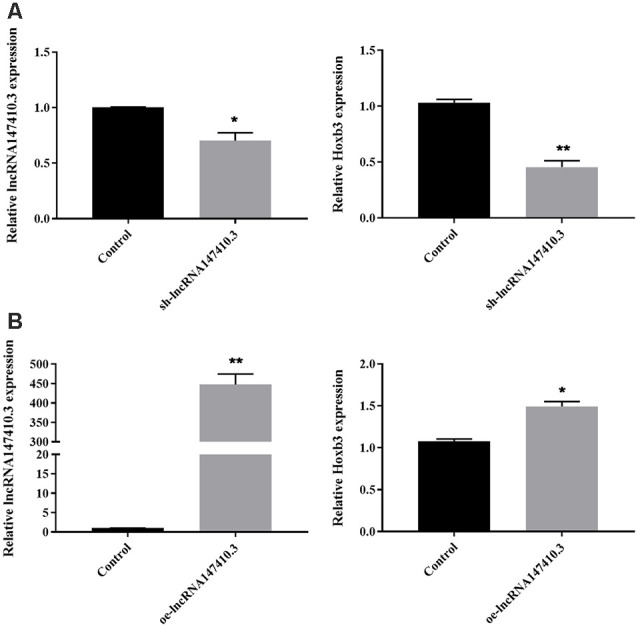
The regulation of lncRNA147410.3 on target gene Hoxb3 was verified in cell level. **(A)** The expression of lncRNA147410.3 and Hoxb3 in BV-2 cells transfected with sh-lncRNA147410.3 or oe-lncRNA147410.3 was tested by qRT-PCR. All values are presented as mean ± SD for three independent experiments. *p < 0.05, **p < 0.01, Student’s *t*-test. **(B)** The expression of lncRNA147410.3 and Hoxb3 in BV-2 cells transfected with oe-lncRNA147410.3 was tested by qRT-PCR. All values are presented as mean ± SD for three independent experiments. *p < 0.05, **p < 0.01, Student’s *t*-test.

To further determine the regulatory relationship of lncRNA147410.3 on the target gene Hoxb3, sh-lncRNA147410.3 and oe-lncRNA147410.3 were transfected into BV-2 cells. The expression of lncRNA147410.3 and Hoxb3 in the BV-2 cells above were quantified by qPCR. The results indicated that the expression of lncRNA147410.3 and Hoxb3 were simultaneously downregulated after transfection with sh-lncRNA147410.3 in BV-2 cells (Figure 2A). Meanwhile, lncRNA147410.3 and Hoxb3 expression were simultaneously upregulated in the cells when the lncRNA147410.3 was overexpressed (Figure 2B). Therefore, it was demonstrated that lncRNA147410.3 can regulate the expression of Hoxb3 positively.

### The Localization of LncRNA147410.3 in the Brain of *T. gondii* Infected Mice

**Figure 3:**
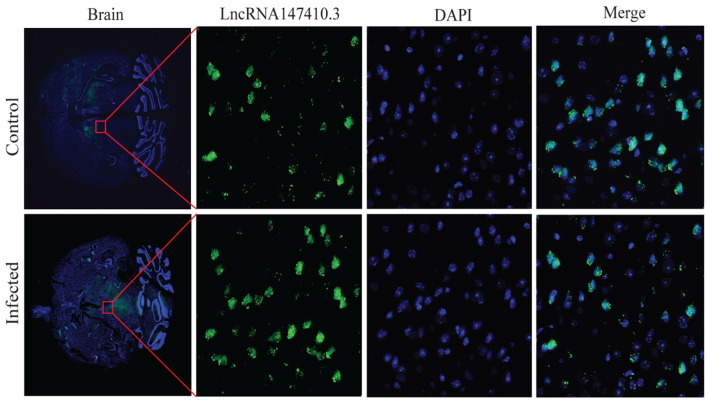
Localization of lncRNA147410.3 expression in the mice brain infected with *T. gondii*. Fluorescence *in situ* hybridization (FISH) binding affinity of lncRNA147410.3 shows a significant downregulation trend in the brain of *T. gondii* infected mice compared with that of the uninfected mice.

To determine the localization of lncRNA147410.3 in the brain tissue, a fluoresce labeled single-stranded lncRNA147410.3 nucleic acid was used as a probe, and localization was observed by confocal microscopy. The photographic results revealed that the fluorescent probe was colocalized with cell nuclei which were stained with DAPI in the brain tissue sections. Thus, lncRNA147410.3 was identified to be localized on the nuclear chromatin of brain, while most of the lncRNAs were mainly clustered in the striatal region in the murine brain (Figure 3).

### The Expression and Localization of LncRNA147410.3 in BV-2 Cells

**Figure 4:**
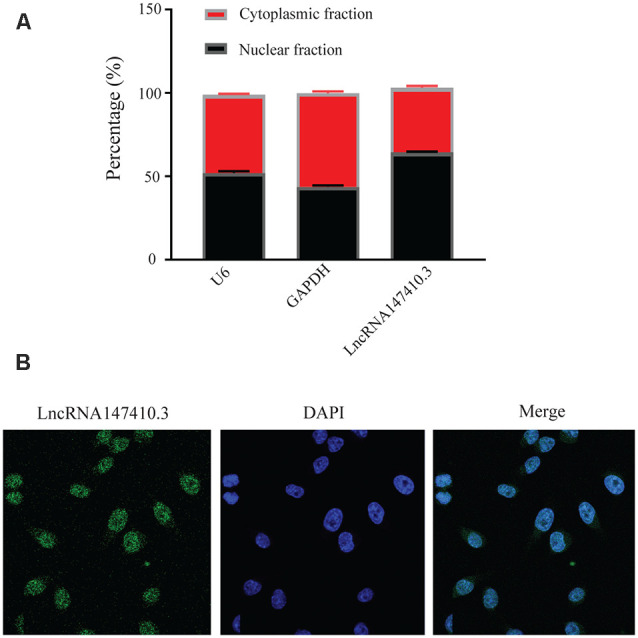
Localization of lncRNA147410.3 in BV-2 cells. **(A)** Nuclear cytoplasmic expression of lncRNA147410.3 in BV-2 cells was detected by qRT-PCR. **(B)** The localization of lncRNA147410.3 in BV-2 cells was detected by FISH assay.

To further clarify the localization of lncRNA147410.3 in neuronal cells, we used BV-2 cells, a type of neuroglia to verify the expression of lncRNA147410.3. qRT-PCR confirmed that the expression of lncRNA147410.3 was significantly higher in the nucleus than in the cytoplasm (Figure 4A). U6 and GAPDH were used as internal reference of nucleus and cytoplasm, respectively. Cellular FISH were also performed to verify that lncRNA147410.3 was distributed mostly in the nucleus of BV-2 (Figure 4B).

### The Effect of LncRNA147410.3 on the Proliferation and Cycle of BV-2 Cells

**Figure 5:**
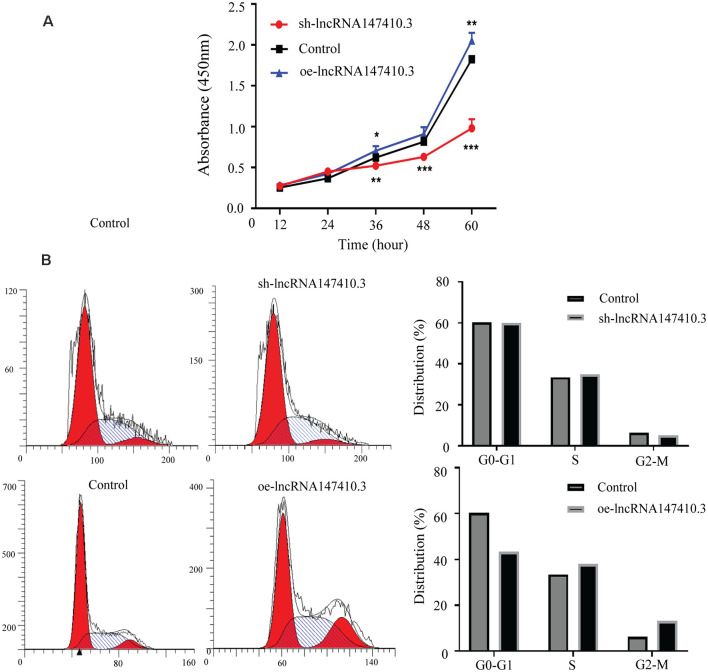
Effects of lncRNA147410.3 on proliferation and cycle of BV-2 cells. **(A)** Cell proliferation was determined by CCK-8 assay which was performed in BV-2 cells transfected with sh-lncRNA147410.3 or oe-lncRNA147410.3 at 12, 24, 36, 48, and 60 h, later. All values are presented as mean ± SD for three independent experiments. *p < 0.05, **p < 0.01, ***p < 0.001, Student’s *t*-*test*. **(B)** The cell cycle of BV-2 cells transfected with sh-lncRNA147410.3 or oe-lncRNA147410.3 was analyzed by Flow cytometry.

To clarify the function of lncRNA147410.3 in the nerve cells, sh-lncRNA147410.3, and oe-lncRNA147410.3 were constructed to investigate the role of lncRNA147410.3 in the cell cycle and proliferation in BV-2 cells. To confirm the effect of lncRNA147410.3 on the proliferation of BV-2 cells, we transfected sh-lncRNA147410.3 and oe-lncRNA 147410.3 plasmids into BV-2 cells, and detected the cell growth at 12, 24, 36, 48, and 60 h of transfection by CCK-8 assay. The results showed that cell proliferation was declined markedly after transfecting with sh-lncRNA147410.3, while cell proliferation was rose significantly after overexpressing lncRNA147410.3 (Figure 5A). Therefore, it was proved that the regulation of lncRNA147410.3 could affect the proliferation of BV-2 cells.

We further verified the effects of lncRNA147410.3 on BV-2 cell cycle after transfection with sh-lncRNA147410.3 and oe-lncRNA147410.3 by flow cytometry. The results showed that when lncRNA147410.3 was overexpressed, the cells number in G0-G1 phases was significantly lower than that of the control group (43.49% vs. 60.30%). This indicates that the upregulation of lncRNA147410.3 can accelerate the G2-M phase of the cell cycle and lead to cell proliferation (Figure 5B).

### The Effect of LncRNA147410.3 on Apoptosis of BV-2 Cells

**Figure 6:**
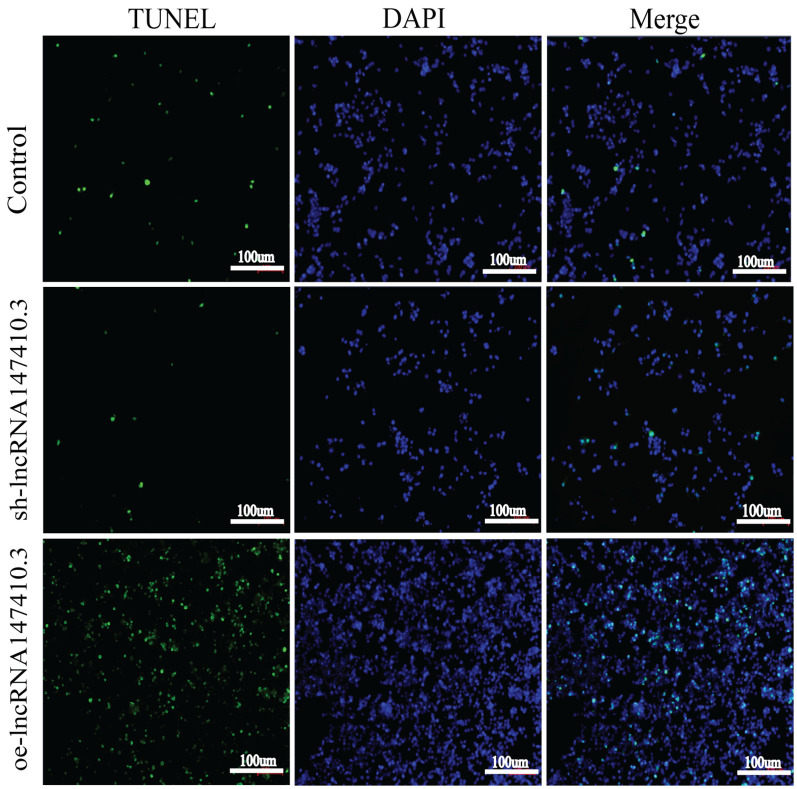
The effect of lncRNA147410.3 on the apoptosis of BV-2 cells was detected by TUNEL assay cell apoptosis of BV-2 cells transfected with control plasmid, sh-lncRNA147410.3 and oe-lncRNA147410.3 was analyzed by TUNEL assay. The coverslips were observed and images are collected by fluorescent microscopy. Apoptosis cells labeled with FITC show green fluorescence. DAPI (blue) was used for counterstaining.

To verify the effect of lncRNA147410.3 on apoptosis of BV-2 cells, we performed TUNEL assay. The results showed that after overexpression of lncRNA147410.3, the fluorescently labeled apoptotic cells were increased significantly compared with the control group, while the fluorescent apoptotic cells in the interference group decreased (Figure 6). Thus, the results indicated that overexpression of lncRNA147410.3 might promote apoptosis in BV-2 cells by targeting Hoxb3. Thus, further certificated that the up-regulation of lncRNA147410.3 in the *T. gondii* infected mice brain could cause apoptosis of microglia.

## Discussion

The role of upregulation of lncRNA147410.3 in *T. gondii* infected mice brain is unclear. In this study, a mouse model of chronic infection was established using *T. gondii* PRU strain. A significant different expression of lncRNAs were detected in the infected mice brain. In the lncRNAs-mRNA microarray, we found that expression level of lncRNA147410.3 was up-regulated significantly. LncRNA147410.3 could affect proliferation, differentiation, and apoptosis of mouse microglia by positively regulating its target gene Hoxb3.

Hoxb3, the target gene of lncRNA147410.3, was predicted by *cis* and *trans* analysis and verified by the qPCR. Hoxb3 is the main member of the Hoxbs family which is expressed in neural stem cells during the early stages of development. It is involved in cerebellar neural stem cells maintenance, and related to the differentiation status of neural cells and oligodendrocyte precursor cells (OPCs; Webster, 2007). We found that the over expression of lncRNA147410.3 would promote the up-regulation of Hoxb3 expression. The up-regulation of Hoxb3 expression in the *T. gondii* infected mice brain may be related to the regulation of microglial function. It has been reported that the primary neurosphere produced by cerebrum nerve stem/progenitor cells are mainly differentiated into astrocytes and oligodendrocytes. Hoxb3 is the main transcription factor regulating the differentiation of oligodendrocytes, which are the myelinating cells of the central nervous system (CNS; De Medeiros Brito et al., 2020). This study first demonstrated that Hoxb3 regulated functional changes in microglia.

The biological functions of lncRNAs are closely related to their subcellular localization, although it is not clear which sites of the specific gene lncRNAs are recruited into. However, lncRNAs located in the nucleus can act as a scaffold for chromatin modifiers by interacting with chromatin modification complexes, or act as transcriptional co-modulators by binding to transcription factors (Naruse et al., 2018). In other words, lncRNA can act as a main regulatory factor on targeted mRNA. LncRNA enriched in the nucleus are involved in key cellular processes including epigenetic regulation, chromosome interaction and transcriptional regulation (Ulitsky and Bartel, 2013). While enriched in cytoplasm, lncRNAs usually participate in post-transcriptional regulation through interaction with microRNA or mRNA. For example, it was found that lncRNA-UCA1 is involved in a typical ceRNA mechanism that influences the progression of colon cancer by forming the lncRNA-UCA1/Mir-28-5p/Hoxb3 axis, culminating in the regulation of gene function expression (Bánfai et al., 2012; Cui et al., 2019). Our study proved that lncRNA147410.3 were concentrated in the corpus striatum of the mice brain and enriched in the nucleus of BV-2 cells based on FISH and qPCR test. This is the first discovery and verification of the location information of lncRNA147410.3. It has been reported that *Toxoplasma* chronic infection can indeed lead to the reduction of probabilistic neural networks (PNNs) in mice. Neural networks embrace the body of neuron and proximal dendrites, which plays a key role in the maintenance and stability of neural circuits. Damage of PNNs leads to abnormal synaptic function and behavioral response (Crapser et al., 2020).

In order to explore the function of lncRNA147410.3 in nerve cells. The effect of lncRNA147410.3 on the proliferation, differentiation, and apoptosis of BV-2 cells was evaluated. We confirmed the relationship between lncRNA147410.3 and Hoxb3 by transfection with overexpression or interference plasmids. We proved that overexpression of lncRNA147410.3 promoted the proliferation and differentiation of BV-2 cells, while knocking down of lncRNA147410.3 inhibited the proliferation and differentiation of BV-2 cells. The increase of G2-M phases cell number in lncRNA147410.3 overexpression BV-2 cell indicated a large amount of RNA and proteins, including tubulins, are synthesized. Thus, the upregulation of lncRNA147410.3 in the *T. gondii* infected mice brain could induce the proliferation and differentiation of the BV-2 cells.

LncRNA regulates many aspects of cell function, affecting gene expression, cell differentiation, apoptosis, and participating in many physiological processes such as inflammation and tumor (Li and Chen, 2016). In our study, through the TUNEL assay, we also found that overexpression of lncRNA147410.3 promoted the apoptosis of microglia cells. Relevant studies have confirmed that lncRNAs can indeed regulate the apoptosis of various cell lines (Elling et al., 2016; Engreitz et al., 2016; Zhang et al., 2018; Wang et al., 2018; Gao et al., 2019). For example, lncRNA-GAS5 in exosomes regulates the apoptosis of macrophages and vascular endothelial cells in atherosclerosis (Chen et al., 2017). LncRNA-h19 promotes the apoptosis of hippocampal neurons by inhibiting the expression of microRNA-let-7b in the epileptic rat model (Han et al., 2018). LncRNA-MEG3 regulates the apoptosis of ischemic neurons by targeting the Mir-21/PDCD4 signaling pathway (Yan et al., 2017). Our study reveals the upregulation of lncRNA147410.3 in the *Toxoplasma* infection mice brain could enhance the apoptosis of the microglia cells. Therefore, the interference of lncRNA147410.3 could be an effective way to rescue this phenomenon and serving as a therapeutic strategy to treat this disease.

In conclusion, in this study, we revealed that the upregulation of lncRNA147410.3 in the brain of mice infected with *T. gondii* can induce cell proliferation, differentiation, and apoptosis by targeting Hoxb3. It could be served as a new mechanism of CNS damage induced by *T. gondii*. Therefore, the findings of this study provide a new experimental basis for a future novel therapeutic strategy for the treatment of this parasitic disease.

## Data Availability Statement

The raw data supporting the conclusions of this article will be made available by the authors, without undue reservation.

## Ethics Statement

The animal study was reviewed and approved by the Code of Ethics for Experimental Animals of Shandong University under Contract LL201902044.

## Authors Contributions

HC designed and supervised the study and revised the manuscript. YW and RH performed the experiments, analyzed the data, and wrote the manuscript. ZX helped to perform the experiments. XS and YW conducted the pathology experiments of the mice. Bioinformatics analysis of lncRNAs, and mRNAs integrated microarrays by CZ. SH helped to design the experiments. BH revised the manuscript. All authors contributed to the article and approved the submitted version.

## Conflict of Interest

The authors declare that the research was conducted in the absence of any commercial or financial relationships that could be construed as a potential conflict of interest.
